# Revival

**Published:** 1863-01

**Authors:** 


### REVIVAL.

The Western Dental Review, has made its appearance again, after a brief suspension. It's one of the things that would not stay suspended. It gives as much pleasure to receive it, and we hope there will be no cause for its suspension in the future. It always comes richly ladened with the sayings and doings of our St. Louis brethren, whom we never think of but to honor, for the work they have done, and the progress they have made, in our noble profession. Vive le Review. T.

